# Webinar: the New Way of Continued Medical Education

**DOI:** 10.1007/s12262-020-02516-0

**Published:** 2020-07-17

**Authors:** Amaresh Biradar

**Affiliations:** Highborn Plastic Surgery Center, Sedam Road, Kalaburagi, Karnataka 585105 India

Sir,

Due to the recent outbreak of the Covid-19 pandemic and nationwide lockdown, there has been an upsurge use of virtual platforms to conduct classes, seminars, webinars, and continued medical education programs (CMEs). These platforms have also been used in every field like cooking classes, yoga classes, fitness, schools, colleges, and businesses. The virtual meeting platforms are also used in surgical fields as well to spread knowledge and science. The virtual platforms have an option of presenting a screen and thereby sharing the PowerPoint presentation. There is a two-way interaction with the audiences happening in real time.

Here, we have various reputed company platforms like Google Meet, [1] Zoom, [2] Microsoft Teams, [3] Cisco Webex, [4] GoToMeeting, [5] YouTube, and Facebook. Platforms like YouTube and Facebook can be used for streaming to the audience and have a one-way video interaction. In these two platforms, the questions can be asked through the comments sections. The other platforms like Google Meet, Zoom, Microsoft Teams, Webex, and GoToMeeting have a two-way discussion with many participants. Most of the platforms need high-speed internet connectivity preferably 4G connections.

Table 1 shows the comparison of the various platforms.

**Table 1:** Comparing various platforms of virtual platforms

	Google Meet [1]	Zoom [2]	Microsoft Teams [3]	Cisco Webex [4]	GoToMeeting [5]
Maxium participants	250	1000 (paid version)	250	200	250
Separate app in mobile	Yes	Yes	Yes	Yes	Yes
Laptop	Chrome browser	Software	Software	Software	Software
User friendly	Easy	Easy	Difficult	Moderate	Easy
Recording option	Yes in paid version	Yes	Yes	Yes	Yes
Stream to YouTube	No	Yes	Yes	Yes	Yes
Subscription price	Free and paid in G-Suite	Rs: 1138 [15.5$]	Rs: 380 [13.5$]	Rs: 1024 [13.5$]	Rs: 910 [12$]

All the features mentioned are as of May 15, 2020. It may change with time.
